# Measuring Viscosity Using the Hysteresis of the Non-Linear Response of a Self-Excited Cantilever

**DOI:** 10.3390/s21165592

**Published:** 2021-08-19

**Authors:** João Mouro, Paolo Paoletti, Michele Basso, Bruno Tiribilli

**Affiliations:** 1 Institute for Complex Systems, National Research Council (ISC-CNR), 50019 Florence, Italy; bruno.tiribilli@isc.cnr.it; 2 School of Engineering, University of Liverpool, Liverpool L69 3GH, UK; P.Paoletti@liverpool.ac.uk; 3 Department of Information Engineering, University of Florence, 50139 Florence, Italy; michele.basso@unifi.it

**Keywords:** microcantilever, viscosity sensing, non-linear dynamics, delay differential equation, hysteresis, bifurcation phenomenon

## Abstract

A self-oscillating microcantilever in a feedback loop comprised of a gain, a saturator, and an adjustable phase-shifter is used to measure the viscosity of Newtonian fluids. Shifting the signal of the loop with the adjustable phase-shifter causes sudden jumps in the oscillation frequency of the cantilever. The exact position of these jumps depends on whether the shift imposed by the phase-shifter is increasing or decreasing and, therefore, the self-excited cantilever exhibits a hysteretic non-linear response. This response was studied and the system modeled by a delay differential equation of motion where frequency-dependent added mass and damping terms accounted for the density and the viscosity of the medium. Experimental data were obtained for solutions with different concentrations of glycerol in water and used to validate the model. Two distinct sensing modalities were proposed for this system: the sweeping mode, where the width of the observed hysteresis depends on the viscosity of the medium, and the threshold mode, where a sudden jump of the oscillation frequency is triggered by an arbitrarily small change in the viscosity of the medium.

## 1. Introduction

Measuring the viscosity of Newtonian and non-Newtonian fluids is crucial in applications such as microfluidics, healthcare, environmental monitoring, and food and process industries. One possible strategy to obtain these measurements is probing the viscous medium with a vibrating mechanical microdevice. Indeed, the dynamic response of such a device is affected by the rheological properties of the surrounding environment [1,2,3] and, therefore, changes observed in the device response can be linked to changes in the fluid properties. Using microscale devices is advantageous to probe smaller time and space scales, allowing measurement of local viscosities (instead of bulk) in real time, with high sensitivities, and using minimal volumes of liquid.

Initial strategies were based on the detection of frequency shifts in the dynamic response of externally excited resonating microdevices, caused by the interaction with the surrounding Newtonian fluid [4,5,6]. Similar strategies to measure the elastic and viscous response of non-Newtonian fluids were also proposed, involving measuring the amplitude and phase of vibrations excited at different frequencies [7,8,9]. However, techniques based on external excitation are limited by the low-quality factor of microresonators’ oscillations in highly viscous medium and by the presence of noise and vibrations coupled with the experimental apparatus (a phenomenon called “forest of peaks” [10,11]). To overcome these limitations, strategies in which the resonator self-oscillates in a feedback loop were subsequently developed [12]. These works typically use microcantilevers or doubly clamped beams, and include delayed force or phase feedback [13,14], *Q*-control [15], or parametric resonance [16,17,18,19] to excite oscillations with very high signal-to-noise ratio. Closed-loop strategies used to measure the viscosity of fluids are shown in [20,21], where a cantilever attached to a disk immersed in a high-viscosity fluid is self-excited with a feedback signal proportional to its velocity.

In particular, a closed-loop feedback system in which the self-oscillations are induced by the competition between a gain and saturator was developed and modeled [22] and subsequently used for atomic force microscopy (AFM) imaging [23]. A controllable analog phase-shifter was then added to this system, and sudden jumps between oscillation frequencies, induced by controlled shifts of the signal in the loop, were observed, modeled [24], and used to measure the viscosity of Newtonian fluids [25].

Recent works described in [26,27] also report the presence of sudden jumps of the oscillation frequencies of self-excited resonators, but showing, in addition, a hysteresis region in the position of these jumps. To study the hysteresis region in [27], the authors develop a self-excited (macro)cantilever by coupling the mechanical dynamics of the cantilever with the electrical dynamics of the piezoelectric layer attached to the cantilever and responsible for its excitation (hence, avoiding using an external displacement detector). They analytically analyzed the fourth-order dynamics of the full system and showed the existence of two Hopf bifurcations by studying the root locus of the eigenvalues of the system. The distance between the two Hopf bifurcation points defines the hysteresis region, whose width depends on the viscosity of the medium. An analogous hysteresis region was also previously observed in [28], where Floquet theory was used to study the stability of an AFM setup used for imaging.

In the present work, new experiments with the setup used in [25] also reveal a similar hysteretic response of the microcantilever. Here, the microcantilever self-oscillates in viscous fluids and the controllable phase-shifter is used to induce the sudden jumps of the oscillating frequency of the system. The exact position of these jumps is observed to depend on whether the shift of the signal in the feedback loop (induced by the adjustable phase-shifter) increases or decreases. It is then proposed to use the sudden jumps and the hysteresis region to enable two new and distinct viscosity-sensing modalities for the cases in which the viscosity of the medium is constant or time-variant. Contrary to the existing setups, the system presented in this work benefits from the self-excitation strategy (overcoming energy losses and showing a very high signal-to-noise ratio), the reduced dimensions and high frequencies of the microcantilever (enabling exploration of smaller space and time scales in real time), and the high sensitivity of the proposed methods, since these are based on sudden jumps of the oscillation frequencies that can be triggered by arbitrarily small changes.

The work is organized as follows. In Section 2, the experimental setup and methodology is presented. Section 3 shows the experimental data and the modeling approach to describe this system. The numerical results obtained were used to propose two different viscosity-sensing modalities. Section 4 discusses some of the limitations of the proposed modeling approach, while Section 5 summarizes the main findings and conclusions of the work.

## 2. Materials and Methods

**Figure 1:**
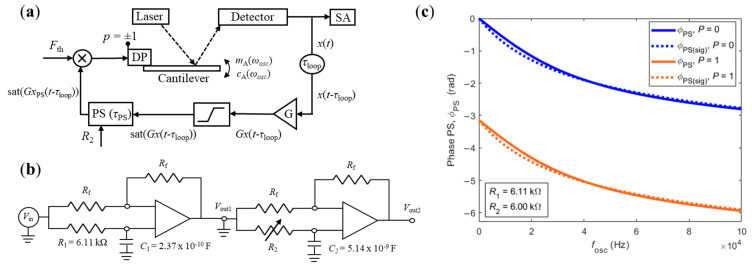
(**a**) Schematic of the experimental setup. The deflection of the cantilever is detected by a four-quadrant detector, naturally delayed, amplified, saturated, controllably delayed with an adjustable Phase-Shifter (PS), and, finally, fed back to the exciting piezo. (**b**) Schematic of the two-stage PS. The second stage was used to control the imposed shift in the feedback loop with the potentiometer *R*_2_. (**c**) Phase-shift introduced by the PS shown in (**b**) as function of the oscillation frequency of the closed-loop given by the transfer function (Equation (2), solid lines) and the sigmoid approximation (Equation (3), dashed lines), for different polarities of the piezo.

The experimental setup considered in this paper is shown in Figure 1a. It consists of a cantilever embedded in a closed-loop feedback system and immersed in a viscous medium. The cantilever was excited by a dither piezo and its motion was detected by reflecting a laser to a four-quadrant detector. This signal was amplified by a gain *G*, then saturated (to avoid exponential growth of the deflection), and, finally, shifted by an adjustable phase-shifter before being fed back to the piezo. The polarity of the signal fed to the piezo could still be changed manually. When closing the feedback loop, the frequency component of the intrinsic thermal noise that satisfies the phase requirement for self-oscillations (total phase-shift of the signal around the feedback loop must be an integer multiple of 2π radians) is amplified, then saturated, until a stable oscillation with frequency $\omega_{osc}=2\pi f_{osc}$ sets in. The frequency of the oscillation is measured by reading the deflection signal with a spectrum analyzer and its amplitude is such that the gain of the loop was unitary [14,24].

The signal from the displacement detector is naturally shifted along the loop, due to the delays caused, for example, by the propagation of the acoustic waves from the piezo to the cantilever tip and through the electronic components [24]. This natural delay is captured in the term $\tau_{loop}$ and causes a natural phase shift of the displacement signal, given by $\phi_{loop}=\omega_{osc}\tau_{loop}$. As discussed in [24,25,29], sudden jumps in the oscillation frequency of the closed-loop occur when the phase of the cantilever is perturbed around its limits (either 0 or −π). In this case, the signal along the feedback loop wraps to a different integer multiple of 2π radians and the phase (and oscillation frequency) of the cantilever jumps to the other edge (−π or 0, respectively).

The Phase-Shifter (PS) included in the experimental setup was used to shift the signal along the feedback loop and perturb the phase (and, therefore, the frequency) of the oscillating cantilever. It consisted of two all-pass filters, connected in series, that could be controlled using two potentiometers, *R*_1_ and *R*_2._ Each stage has its own capacitance (*C*_1_ = 2.37 × 10^−10^ F and *C*_2_ = 5.14 × 10^−9^ F) and works effectively in a different range of frequencies (typically associated with the oscillation of the cantilever in air or liquid, respectively). The transfer function of the PS shown in Figure 1b is given by

(1) $$PS\left(j\omega \right)=\frac{V_{out2}}{V_{in}}=\frac{V_{out2}}{V_{out1}}\times \frac{V_{out1}}{V_{in}}\times p=\frac{1-j\omega R_{1}C_{1}}{1+j\omega R_{1}C_{1}}\times \frac{1-j\omega R_{2}C_{2}}{1+j\omega R_{2}C_{2}}\times p,$$

with p=± 1, depending on the polarity of the signal fed to the piezo. The phase-shift introduced by the adjustable PS, $\phi_{\mathrm{PS}}$, is then given by

(2) $$∠PS\left(j\omega \right)=\phi_{\mathrm{PS}}=-2\mathrm{atan}\left(\omega R_{1}C_{1}\right)-2\mathrm{atan}\left(\omega R_{2}C_{2}\right)-P\pi ,$$

where P=1 when p=−1 and P=0 when *p* = 1. Note that the adjustable $\phi_{\mathrm{PS}}$ added to the natural phase shift of the loop, $\phi_{loop}$, mentioned previously. As discussed in Section 3.2.1, it is helpful to approximate the phase-shift introduced by the PS, $\phi_{\mathrm{PS}}$, given by Equation (2), via a sigmoid as

(3) $$\phi_{\mathrm{PS}}\approx \phi_{\mathrm{PS}\left(sig\right)}=-\pi \frac{\omega R_{1}C_{1}}{1+\omega R_{1}C_{1}}-\pi \frac{\omega R_{2}C_{2}}{1+\omega R_{2}C_{2}}-P\pi .$$

The phase-shift introduced by the adjustable PS in the feedback loop is a function of the oscillation frequency of the loop, $\omega_{osc}$. Figure 1c shows a comparison between the phase-shifts predicted by Equations (2) and (3) for different polarities applied to the piezo and constant values of *R*_1_ and *R*_2_ (used in this work). As can be observed, the approximation used in Equation (3) introduces negligible errors.

## 3. Results

### 3.1. Experimental Measurements

All the experimental results presented in this paper were obtained using a standard tipless ACST-TL cantilever from AppNano, with nominal length *L* = 160 µm, width *W* = 33 µm, and thickness *T* = 2.5 µm. The natural frequency and quality factor in air were estimated as, respectively, *f*_0_ = 139.4 kHz and *Q* = 240, by sweeping the frequency of a classical external excitation scheme and fitting a Lorentzian curve to the measured frequency response.

The experimental protocol used to understand the system behavior in viscous solutions consisted of fixing the polarity and *R*_1_ of the PS and then sweeping the value of the potentiometer *R*_2_ up and down, while recording the oscillation frequencies of the closed-loop. Four different solutions of water and glycerol were used: (1) pure water, (2) water + 5% glycerol (*v*/*v*), (3) water + 10% glycerol (*v*/*v*), and (4) water + 15% glycerol (*v*/*v*). These corresponded to medium viscosities of, respectively, 1.005 × 10^−3^, 1.239 × 10^−3^, 1.384 × 10^−3^, and 1.650 × 10^−3^ Pa s at 20 °C [30]. The density of the water–glycerol solutions does not change significantly with the concentration of glycerol (only ~2.5 % in this range [30]) and, therefore, the solution density was assumed to be constant and equal to the density of water (998 kg/m^3^ at 20 °C [30]) throughout this work.

Constant values of the potentiometer *R*_1_ (*R*_1_ = 6.11 kΩ) and polarity (*p* = −1 with *P* = 1) were chosen so that sudden jumps in oscillation frequency were observed when sweeping *R*_2_ [24,27].

**Figure 2:**
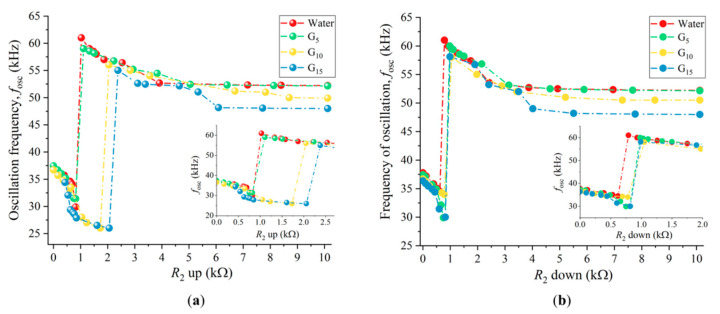
Experimental oscillation frequencies of the self-excited microcantilever as function of the phase-shift introduced by the PS, with fixed *R*_1_ and polarity (*p* = −1). (**a**) Potentiometer *R*_2_ swept up; (**b**) potentiometer *R*_2_ swept down. Insets detail the jump regions and G_5_, G_10,_ and G_15_ indicate the concentration of the glycerol solutions.

Figure 2 shows the experimental results obtained when sweeping the potentiometer *R*_2_ up and down while the cantilever was immersed in the four viscous solutions. It was observed that when *R*_2_ was swept up (Figure 2a), the position of the sudden jump from low to high frequencies changed with the viscosity of the medium. Higher viscosities required a higher value of *R*_2_ (bigger phase-shift imposed by the PS on the feedback loop) to jump.

When sweeping *R*_2_ down (Figure 2b), this dependence was less evident, and the sudden jump from high to low frequencies occurred for similar values of *R*_2_. Furthermore, it was observed that the position of the jumps from low to high frequencies (increasing *R*_2_) and from high to low frequencies (decreasing *R*_2_) did not match. This defined an hysteresis region delimited by two bifurcations, as also found in [27].

### 3.2. Modeling of the System Behavior

#### 3.2.1. Equation of Motion

The response of the experimental setup shown in Figure 1a can be modeled by a delay differential equation of the single-degree-of-freedom damped-harmonic oscillator, i.e.,

(4) $$\left(m_{0}+m_{A}\left(\omega_{osc}\right)\right)\ddot{x}\left(t\right)+\left(c_{0}+c_{A}\left(\omega_{osc}\right)\right)\dot{x}\left(t\right)+k_{1}x\left(t\right)=\left[\mathrm{sat}\left(Gx_{\mathrm{PS}}\left(t-\tau_{loop}\right)\right)\right],$$

where x(t) is the deflection of the cantilever in time and the dots are its time derivatives, $m_{0}=\rho LWT$ is the total mass of the cantilever, $c_{0}=\frac{\omega_{0}m_{0}}{Q}$ is the intrinsic damping coefficient (with $\omega_{0}$ and Q as the natural frequency and intrinsic quality factor of the first resonance mode of the cantilever), $m_{A}\left(\omega_{osc}\right)$ and $c_{A}\left(\omega_{osc}\right)$ are the added mass and damping coefficients due to the viscous fluid (with $\omega_{osc}$ as the oscillation frequency of the system), and $k_{1}=\frac{EWT^{3}}{4L^{3}}\frac{3}{{\left(\beta_{1}L\right)}^{4}}$ is the effective spring constant of the first flexural mode (with $\beta_{1}L=1.875,\;\frac{3}{{\left(\beta_{1}L\right)}^{4}}=0.243$ [31,32,33], and *E* = 180 GPa as the Young’s modulus of silicon). The right side of Equation (4) describes the force that acts on the cantilever, where sat() represents the saturation function, G represents the gain, $\tau_{loop}$ represents the natural delay along the loop and $x_{\mathrm{PS}}$ represents the output of the PS (dependent on the chosen values of the *R*_1_, *R*_2,_ and on the oscillation frequency of the closed-loop $\omega_{osc}$).

The added mass and damping terms $m_{A}$ and $c_{A}$ induced by the fluid on the vibrating cantilever correspond to the inertial and viscous parts of the hydrodynamic function developed by Sader and Maali [1,3], i.e.,

(5) $$m_{A}\left(\omega_{osc}\right)=\frac{\pi }{4}\rho_{f}W^{2}\left(a_{1}+\frac{a_{2}}{W}\sqrt{\frac{2\eta }{\rho_{f}\omega_{osc}}}\right),$$

(6) $$c_{A}\left(\omega_{osc}\right)=\frac{\pi }{4}\rho_{f}W^{2}\omega_{osc}\left(\frac{b_{1}}{W}\sqrt{\frac{2\eta }{\rho_{f}\omega_{osc}}}+\frac{b_{2}}{W^{2}}\frac{2\eta }{\rho_{f}\omega_{osc}}\right),$$

where $\omega_{osc}$ is the oscillation frequency of the system, $\rho_{f}$ and η are the density and viscosity of the fluid, respectively, and *a*_1_ = 1.0553, *a*_2_ = 3.7997, *b*_1_ = 3.8018, and *b*_2_ = 2.7364.

The output signal of the PS, $x_{\mathrm{PS}}$, can be related with the deflection of the cantilever, x, if the PS is approximated by a pure delay with $\tau_{\mathrm{PS}}$ (see Figure 1a). In this case, the right hand of Equation (4) can be rewritten as

(7) $$\mathrm{sat}\left(Gx_{\mathrm{PS}}\left(t-\tau_{loop}\right)\right)\approx \mathrm{sat}\left(Gx\left(t-\tau_{loop}-\tau_{\mathrm{PS}}\right)\right).$$

Under this framework, the delay introduced by the PS, $\tau_{\mathrm{PS}}$, is a function of the polarity and the values of the potentiometers *R*_1_ and *R*_2_ and can be modeled using Equation (3). In this case, it was assumed that the delay $\tau_{\mathrm{PS}}$ is the proportionality constant between the phase-shift that it introduces in the feedback loop (given by Equation (3)) and its oscillation frequency $\omega_{osc}$, as

(8) $$\tau_{\mathrm{PS}}\left(\omega_{osc}\right)=-\frac{\phi_{\mathrm{PS}\left(sig\right)}}{\omega_{osc}}=\frac{\pi }{\frac{1}{R_{1}C_{1}}+\omega_{osc}}+\frac{\pi }{\frac{1}{R_{2}C_{2}}+\omega_{osc}}+P\frac{\pi }{\omega_{osc}}.$$

Substituting Equation (7) into Equation (4), the final equation of motion is written as

(9) $$\left(m_{0}+m_{A}\left(\omega_{osc}\right)\right)\ddot{x}\left(t\right)+\left(c_{0}+c_{A}\left(\omega_{osc}\right)\right)\dot{x}\left(t\right)+k_{1}x\left(t\right)=\mathrm{sat}\left(Gx\left(t-\tau_{loop}-\tau_{\mathrm{PS}}\left(\omega_{osc}\right)\right)\right),$$

where the terms $m_{A}\left(\omega_{osc}\right)$, $c_{A}\left(\omega_{osc}\right),$ and $\tau_{\mathrm{PS}}\left(\omega_{osc}\right)$ are described by Equations (5), (6), and (8), respectively. All these terms depend on the oscillation frequency of the loop to be determined, $\omega_{osc}$. In addition, $m_{A}\left(\omega_{osc}\right)$ and $c_{A}\left(\omega_{osc}\right)$ also depend on the viscosity and density of the medium. The $\tau_{loop}$ is a constant of the feedback loop and can be experimentally determined, as in [24], by measuring the input/output transfer function of each element of the circuit.

The highly non-linear nature of Equation (9) prevents obtaining analytical solutions to determine the oscillation frequency of the self-excited cantilever, and, therefore, the numerical approach described below was proposed to perform the analysis.

#### 3.2.2. Solving for the Oscillation Frequency of the Loop, *ω_osc_*

By defining $x_{2}\left(t\right)=x\left(t\right)$ and $x_{1}\left(t\right)={\dot{x}}_{2}\left(t\right)=\dot{x}\left(t\right)$, Equation (9) can be written as a system of first-order delay differential equations as

(10) $$\left\{ \begin{array}{c} {\dot{x}}_{1}\left(t\right)=-\frac{c_{0}+c_{A}\left(\omega_{osc}\right)}{m_{0}+m_{A}\left(\omega_{osc}\right)}x_{1}\left(t\right)-\frac{k_{1}}{m_{0}+m_{A}\left(\omega_{osc}\right)}x_{2}\left(t\right)+B\;\tanh\left(Gx_{2}\left(t-\tau_{loop}-\tau_{\mathrm{PS}}\left(\omega_{R}\right)\right)\right),\\ {\dot{x}}_{2}\left(t\right)=x_{1}\left(t\right) \end{array}\right.$$

where the saturator is also approximated by a (naturally limited) continuous hyperbolic tangent function multiplied by a constant *B*. A constant oscillation frequency is imposed in the delay induced by the PS in Equation (10), defined by $\tau_{\mathrm{PS}}\left(\omega_{R}\right)=\frac{\pi }{\frac{1}{R_{1}C_{1}}+\omega_{R}}+\frac{\pi }{\frac{1}{R_{2}C_{2}}+\omega_{R}}+P\frac{\pi }{\omega_{R}}$, where a constant $\omega_{R}$ is used in all terms. This approximation will be justified in Section 4, but it is used to further simplify the problem and improve the numerical stability of the solver. In practice, this approximation makes the delay introduced by the PS, $\tau_{\mathrm{PS}}\left(\omega_{R}\right)$, dependent on the variable parameter *R*_2_ only (*P*, *R*_1,_ and $\omega_{R}$ are fixed in the model).

Delay differential equations with constant delays, such as Equation (10), can be solved with the numerical solver *dde23* of Matlab by providing a past history of the function and integrating for a chosen period with a variable-step Runge–Kutta method (see [34] for details). Solving the system of Equation (10) for $\omega_{osc}$ requires an initial estimation of the added mass and damping terms (Equations (5) and (6)), using the values of the density and viscosity of the medium, $\rho_{f}$ and η, and choosing an initial frequency ($\omega_{i}$, ideally close to the expected final oscillation frequency). Subsequently, the polarity *P* and the values of the potentiometers *R*_1_ and *R*_2_ are fixed, resulting in a constant $\tau_{\mathrm{PS}}\left(\omega_{R}\right)$. Finally, the system of Equation (10) is integrated for a chosen time interval (4 ms), with $x_{1}\left(t\right)=0$ and $x_{2}\left(t\right)=0.1$ when $\left(-\tau_{loop}-\tau_{\mathrm{PS}}\left(\omega_{R}\right)\right)\leq t\leq 0$, as the past history of the function. The solution from the solver is interpolated with the Matlab function *deval* to obtain evenly time-spaced results (time step of 10^−8^ s). Finally, the oscillation frequency of the system is obtained by detecting the maximum of the Power Spectral Density (PSD), calculated from the Fast Fourier Transform (FFT) of the time deflection signal $x_{2}\left(t\right)$ after transients are removed.

The oscillation frequency obtained after solving the system of Equation (10) once (first iteration, $\omega_{osc\_1}$) is already significantly different from the initial frequency used to initialise the added mass and damping terms ($\omega_{i}$). For full consistency, an iterative process can be implemented where Equation (10) can then be solved again using the value of $\omega_{osc\_1}$ to initialise the added mass and damping coefficients, and the analysis repeated to obtain a new frequency of the loop, $\omega_{osc\_2}$ (second iteration), and so on. Nevertheless, it was observed that the consecutively measured oscillation frequencies converged very fast and that solving the system of Equation (10) once was already enough to obtain a constant value for the oscillation frequency of the loop, i.e., $\omega_{osc\_1}\approx \omega_{osc\_2}\approx \omega_{osc}$.

**Figure 3:**
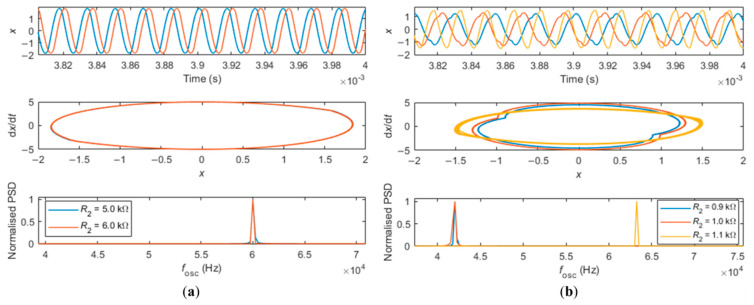
Solving system of Equation (10) for different values of increasing *R*_2_, for the case of the cantilever oscillating in water. (**a**) Top, middle, and bottom rows show the time deflection, phase space, and normalized Power Spectral Density (PSD) of the signals far from the jump region for different values of *R*_2_, respectively. (**b**) Time deflection, phase space, and normalized Power Spectral Density (PSD) of the signals close to the jump region, for different values of *R*_2_.

**Figure 4:**
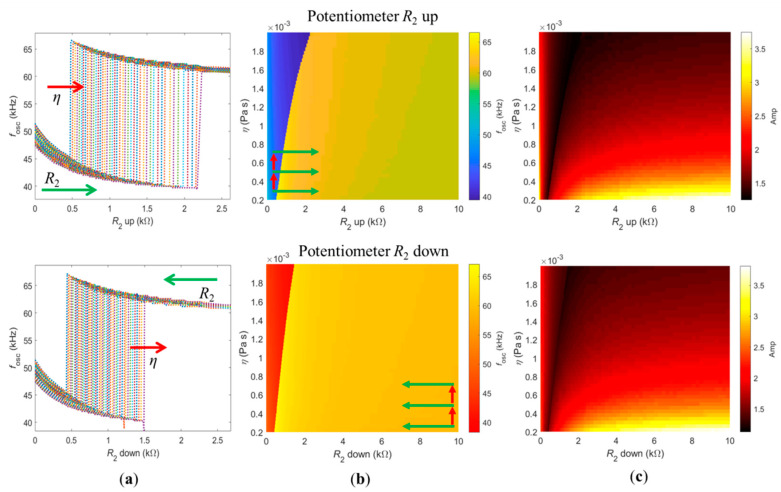
Dependence of the oscillation frequencies on the viscosity of the medium and value of *R*_2_ when the potentiometer was swept up (upper row) or swept down (lower row). (**a**) Jump region when sweeping *R*_2_, for different viscosities. (**b**) Color map of the oscillation frequencies and (**c**) color map of the amplitude of oscillation, for the full range of potentiometer and viscosities used.

**Figure 5:**
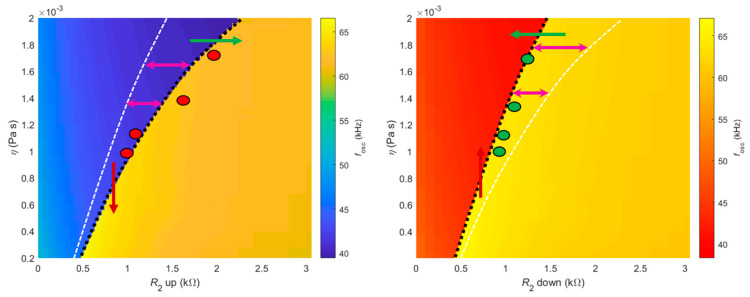
Left and right panels show insets of the oscillation frequency map when *R*_2_ was swept up and down, respectively (Figure 4b, top and bottom rows), with thick, dashed, black lines delimiting the jumps. The red and green circles represent the experimental data measured and presented in Figure 2a,b, for the four glycerol solutions. The thin, white, dashed lines represent the jump delimitation of the opposite panel, for an easier visualization of the two distinct sensing modalities proposed.

When solving the system of Equation (10) and showing the results in Figure 3, Figure 4 and Figure 5, a frequency $\omega_{R}$ in the interval between the high- and low-frequency branches ($\omega_{R}=2\pi \times 55,000$ rad/s) and a closed-loop delay $\tau_{loop}=10.7\;\mu s$ were fixed. The value of $\tau_{loop}=10.7\;\mu s$ used in the simulations compare well with the value of $\tau_{loop\_exp}=8.9\;\mu s$ measured experimentally in [24].

Figure 3 illustrates the process for determining the frequency of oscillation of the loop, $\omega_{osc}$, as detailed in the previous paragraph, for the case of a cantilever oscillating in water, with *R*_2_ swept up (red line and symbols of Figure 2a).

Figure 3a shows results when sweeping *R*_2_ in the region away from the jump (*R*_2_ = 5 kΩ and *R*_2_ = 6 kΩ, see Figure 2a). Increasing *R*_2_ delays the time displacement signal (top row), but the phase space ($x_{1}\left(t\right)$ vs. $x_{2}\left(t\right)$) and oscillation frequency remain essentially unaltered (middle and bottom rows, respectively). Figure 3b shows the case around the jump region (*R*_2_ = 0.9 kΩ, *R*_2_ = 1.0 kΩ and *R*_2_ = 1.1 kΩ, see Figure 2a). Here, it can be observed that the time displacements in this region are no longer described by pure sinusoids, but that the motion already contains components at different frequencies (top row, blue and orange curves), due to the eminency of the sudden jump between different oscillation frequencies (see also [24]). By increasing *R*_2_, a sudden change in the time displacement signal occurs (yellow curve, *R*_2_ = 1.1 kΩ), which corresponds to the jump to a higher frequency of oscillation. This jump can also be seen in the sudden change of the shape of the phase space (middle row) and by the normalized PSD curves (bottom row).

### 3.3. Simulation Results

#### 3.3.1. Dependence of the Oscillation Frequency with Viscosity and Potentiometer *R*_2_

The process for determining the self-oscillation frequency of the closed-loop system described in Section 3.2.2 was then used to study its dependence on the viscosity of the medium, while sweeping *R*_2_ up and down. The results are shown in Figure 4. In these simulations, the viscosity of the medium was varied between *η* = 0.2 × 10^−3^ Pa s and *η* = 2.0 × 10^−3^ Pa s, in steps of *η* = 0.04 × 10^−3^ Pa s, while *R*_2_ was increased or decreased between 0 and 10 kΩ, in variable steps (narrower in the jump region), for constant *R*_1_ = 6.11 kΩ and polarity *p* = −1 (*P* = 1).

The top row shows the case of increasing *R*_2_. The simulation proceeded row by row, with constant viscosity, while *R*_2_ was swept up. The added mass and damping coefficients were initialized with the frequency of the previous calculated point, then the value of *R*_2_ was incremented, and the system of Equation (10) was solved for the new oscillation frequency. This method proceeded until the potentiometer *R*_2_ was fully swept, as indicated by the horizontal green arrows in the top row of Figure 4b. After the complete sweeping of *R*_2_, the viscosity of the system was increased. In this case, the added mass and damping coefficients were initialized with the oscillation frequency and *R*_2_ of the first point of the previous viscosity row. Then, the value of viscosity was incremented and the system of Equation (10) was solved to determine the oscillation frequency of the first point of the new viscosity row, as indicated by the red arrows in Figure 4b. The simulation protocol was the same for the case of decreasing *R*_2_, as indicated by the colored arrows in Figure 4b, bottom row.

Figure 4a shows a clear dependence between the value of potentiometer *R*_2_ required to jump from low to high (top row) and from high to low frequencies (bottom row) with the viscosity of the medium. Nevertheless, the position of the sudden frequency jump was less sensitive to the viscosity when *R*_2_ was swept down (bottom row). This frequency dependence is color-mapped in Figure 4b, with the jump from low to high frequencies (increasing *R*_2_, top row) delimited by the line between the blue and yellow areas, and the jump from high to low frequencies (decreasing *R*_2_, bottom row) delimited by the line between the yellow and red areas.

Figure 4c shows the amplitude of the oscillation for each condition, measured directly from the deflection curves shown in the top panels of Figure 3. The jump between frequency branches is also evident in the amplitude map, and it appears as a delimiting line with minimum amplitude, as indicated by the darkest color. This is expected since the jumps between low and high frequencies correspond to jumping between the limits of the amplitude/phase curves of the cantilever frequency response [29]. Away from the jump, higher values of potentiometer *R*_2_ causes a higher amplitude of deflection since the oscillation frequency of the loop gets closer to the natural frequency of the cantilever. Higher amplitude deflection is also observed in low-viscosity mediums, which can be explained by the reduced damping induced by the cantilever–fluid interaction. Note that the magnitude of the values of amplitude of oscillation are arbitrary and depend on the chosen values of *B* and *G* (see Equation (10)) used in the simulations.

#### 3.3.2. Sensing Modalities

Figure 5 highlights insets of the Figure 4b. The left panel of Figure 5 shows the jump from low to high frequencies (increasing *R*_2_) and the right panel of Figure 5 shows the jump from high to low frequencies (decreasing *R*_2_). The red and green circles indicate the values of potentiometer *R*_2_ for which the jumps were experimentally registered for each solution (different viscosities), as shown in Figure 2a,b, respectively. The thick, dashed, black lines delimit the jump region while the thin, white lines represent the jump region of the opposite panel.

Figure 5 is useful to discuss two different sensing modalities proposed for the device described in this paper. The first, termed sweeping mode, consists of progressively sweeping the potentiometer *R*_2_ up and down, while the self-excited cantilever is immersed in a solution of constant density and viscosity. By measuring the values of *R*_2_ required for the first jump, from low to high frequency (sweeping up), and for the second jump, from high to low frequency (sweeping down), one can then determine the viscosity. Indeed, the difference between these values, or the width of the hysteresis, is univocally connected to the viscosity of the medium, as shown by the purple double arrows in both panels. The width of the hysteresis increases (non-linearly) with the viscosity of the medium. The second working modality is termed threshold mode. In this case, the sensor should be self-oscillating in a solution whose viscosity changes with time. It is this change in the viscosity of the medium that triggers the jump between oscillation frequencies.

Note that the exact same hysteresis region defined by the area between the dashed black and white lines in both panels was defined when viscosity increased/decreased. When the viscosity of the medium decreases, the system follows the behavior indicated on the left panel (corresponding to *R*_2_ swept up), jumping from low to high frequencies. This is shown by the decreasing red arrow on the left panel. Conversely, if the viscosity of the medium increases, the system follows the behavior indicated in the right panel (*R*_2_ swept down), jumping from high to low frequencies. This is also indicated by the respective red arrow on the right panel.

## 4. Discussion

A detailed mathematical model was developed to explain the dynamic response of the self-excited microcantilever, exhibiting sudden jumps of the oscillation frequency and the existence of a hysteresis region, and shed some light on the physics behind it. Such a model was successfully validated with experimental data and can easily be extended to other ranges of viscosities or geometries.

A simplifying assumption used in the proposed model was to impose a constant frequency in the delay imposed by the PS, $\tau_{\mathrm{PS}}\left(\omega_{R}\right)$, in the system of Equation (10). This allowed $\tau_{\mathrm{PS}}\left(\omega_{R}\right)$ to depend on the value of *R*_2_ only and to clearly separate between the jumps in frequency when *R*_2_ was swept up or down, defining the hysteresis region.

However, observing Equation (8) for $\tau_{\mathrm{PS}}\left(\omega_{osc}\right)$, one notes that there are two competing effects that play a role if the value of *R*_2_ and the oscillation frequency $\omega_{osc}$ are simultaneously updated. When sweeping *R*_2_ up with constant $\omega_{osc}$, then $\tau_{\mathrm{PS}}$ increases. If $\tau_{\mathrm{PS}}$ gets bigger than the threshold value required to jump from low to high frequencies, the jump occurs. However, on the other hand, if the jump occurs, the oscillation frequency $\omega_{osc}$ suddenly increases (with constant *R*_2_) and, therefore, $\tau_{\mathrm{PS}}$ suddenly decreases. In this case, $\tau_{\mathrm{PS}}$ may become again smaller than the threshold value required to jump from low to high frequency, and the system will go back to the low-branch solution (the reasoning is equivalent but opposite when *R*_2_ is swept down). In summary, when simultaneously updating *R*_2_ and $\omega_{osc}$ in $\tau_{\mathrm{PS}}\left(\omega_{osc}\right)$ of Equation (8), the solution of Equation (10) jumps back and forth between the two solution branches. The hysteresis region is then measured as the *R*_2_ interval that causes the system to alternate between the two branches. Above a certain value of *R*_2_, the system remains definitely in the upper solution branch, since *R*_2_ gets sufficiently big (high $\tau_{\mathrm{PS}}$) to guarantee that the decrease in $\tau_{\mathrm{PS}}$ that occurs when the system jumps to the high-solution branch does not get lower than the threshold value. This mechanism allows us to conjecture that the system shows two fold bifurcations, leading to the sudden jumps and defining the hysteresis region.

Finally, if $\omega_{R}$ in $\tau_{\mathrm{PS}}\left(\omega_{R}\right)$ is chosen in the middle of the frequency interval between branches ($\omega_{R}=2\pi \times 55,000$ rad/s in this work), the simulation predictions are the same as if updating $\omega_{osc}$. If some other $\omega_{R}$ was chosen, then $\tau_{loop}$ could still be adjusted to displace the hysteresis region to match the experimental data.

As already stated, fixing $\omega_{R}$ in $\tau_{\mathrm{PS}}\left(\omega_{R}\right)$ removed the dependence on the oscillation frequency, $\omega_{osc}$, from the delay induced by the PS. Therefore, the hysteresis phenomenon was solely captured by the dependence of the added mass and damping coefficients on the oscillation frequency $\omega_{osc}$ and η. As discussed in Figure 5, increasing *R*_2_ or decreasing the viscosity η is physically equivalent, since the jumps from low to high frequencies follow the same path. A physical interpretation of this phenomenon is based on the following observation: An increase on either $\omega_{osc}$ or η is responsible for an increase of the ratio between Equations (5) and (6), $c_{A}\left(\omega_{osc}\right)/m_{A}\left(\omega_{osc}\right)$. So, assuming that the system is oscillating in the low-frequency branch and that viscosity increases, then $\eta ↑\;\overset{}{\Rightarrow }c_{A}\left(\omega_{osc}\right)/m_{A}\left(\omega_{osc}\right)↑$. On the other hand, if *R*_2_ decreases, then the delay imposed by the PS in the loop also decreases. This forces the cantilever to compensate, by increasing its phase and, consequently, the oscillation frequency of the loop and the ratio $c_{A}\left(\omega_{osc}\right)/m_{A}\left(\omega_{osc}\right)$, or, schematically: $R_{2}↓\;\overset{}{\Rightarrow }\tau_{\mathrm{PS}}↓\overset{}{\Rightarrow }\phi_{\mathrm{CT}}↑\overset{}{\Rightarrow }\omega_{osc}↑\overset{}{\Rightarrow }c_{A}\left(\omega_{osc}\right)/m_{A}\left(\omega_{osc}\right)↑$.

This shows the equivalence of decreasing *R*_2_ or increasing the viscosity η (the conclusions are opposite when the system is oscillating in the high-solution branch and viscosity decrease or *R*_2_ increase, as also seen in Figure 5).

The physical mechanisms inducing the observed hysteresis are linked to the non-linear behavior of the terms modeling the cantilever–fluid interaction, namely, $m_{A}$ and $c_{A}$. Indeed, as stated above, increasing $\omega_{osc}$ increases the ratio $c_{A}\left(\omega_{osc}\right)/m_{A}\left(\omega_{osc}\right)$, which, in turn, decreases the quality factor of the oscillation, $Q_{R}$. Therefore, a jump from the low-frequency branch to the high-frequency branch is accompanied by a reduction of the quality factor of the oscillation (and vice versa), which indicates that the jumps require some energy transfer between the vibrating cantilever and the surrounding fluid to occur, to accommodate the sudden change of displaced fluids and drag. This mechanism depends on whether the frequency increases or decreases, which gives rise to the hysteresis.

## 5. Conclusions

A new setup to measure viscosity of Newtonian fluids was presented, modeled, and discussed in this paper. The proposed sensing platform is based on the non-linear dynamic response of a microcantilever embedded on a feedback loop exhibiting sudden jumps of oscillation frequency, which define a hysteresis region. It is proposed that the dynamic response of this system can be exploited for two distinct sensing modalities, depending on whether the viscosity of the medium is constant (sweeping mode) or changing in time (threshold mode). In the sweeping mode, viscosity of the fluid can be accurately measured by detecting frequency jumps induced by user-controlled changes in the phase-shifter. On the other hand, the threshold-mode sensing modality can, in principle, detect arbitrarily small changes in viscosity in real time, which can be used, for example, to monitor real-time chemical reactions where a threshold value of some analyte or reagent must be detected. The detection of sudden jumps in the oscillation frequency of the microcantilever does not depend on classical sources of noise, such as the signal-to-noise ratio of the four-quadrant detector, or on the quality factor of the resonance (as in typical setups where frequency shifts are detected), and, therefore, is promising for obtaining improved sensitivity.

## Data Availability

The data presented in this study are available on request from the corresponding author.
